# A Consultation for Pediatric Neck Mass Resulting in a Rare Diagnosis of Klippel-Feil Syndrome: A Case Report

**DOI:** 10.7759/cureus.48579

**Published:** 2023-11-09

**Authors:** Scott McClintick, Kent McIntire, Kindall Martin, Randall Hansen, Hanen Rojas, Christopher Stewart, Suporn Sukpraprut-Braaten

**Affiliations:** 1 Otolaryngology - Head and Neck Surgery, Freeman Health System, Joplin, USA; 2 Medicine, Kansas City University of Medicine and Biosciences, Joplin, USA; 3 Graduate Medical Education, Kansas City University of Medicine and Biosciences, Joplin, USA

**Keywords:** pediatric neck mass, congenital, cervical fusion, neck mass, klippel-feil syndrome

## Abstract

Klippel-Feil syndrome (KFS) is a rare congenital cervical vertebrae fusion syndrome characterized by the clinical triad of low posterior hairline, limited head and neck range of motion, and short neck. The gene defects described with this syndrome are involved in the maturation and differentiation of bone during embryological development. As such, related defects seen in patients with KFS include genitourinary anomalies, cardiac defects, neurological abnormalities, and other musculoskeletal anomalies. Patients with this syndrome should be worked up for these related malformations, evaluated for risk of comorbidities, and educated on avoiding contact sports or activities that may put the cervical spine at risk for trauma.

The case report here describes a pediatric patient who presents to the outpatient otolaryngologist complaining of a pediatric neck mass. Workup of the mass, including imaging, revealed a diagnosis of Klippel-Feil syndrome. The presentation of this case highlights the importance of maintaining KFS on the list of possible diagnoses along with scoliosis, synostosis syndrome, and Wildervanck syndrome for the otolaryngologist working up a neck mass and the role making an early diagnosis can have in preventing patient morbidity and mortality.

## Introduction

First described in 1912 by Maurice Klippel and Andre Feil, Klippel-Feil syndrome (KFS) is a rare genetically inherited disorder characterized by congenital fusion of the cervical vertebrae [1-9]. Initially described with the clinical triad of low posterior hairline, short neck, and limited range of motion of the head and neck, subsequent studies have found that less than 50% of patients with KFS exhibit this triad [2-9]. Klippel-Feil syndrome has an estimated reported incidence of about one in 40,000-42,000 live births, with females making up 60% of these cases [1-3,5,7,9,10]. However, recent literature shows that the prevalence of KFS is much higher and often goes undiagnosed (as high as one in 172 births) [10]. Many of these cases were incidentally found during imaging studies, and patients were asymptomatic [10]. Patients with this syndrome are at an increased risk for spinal injuries, even from minor trauma, making early diagnosis an important aspect of morbidity and mortality prevention in patients with KFS [2,3,7,8,10].

## Case presentation

**Figure 1:**
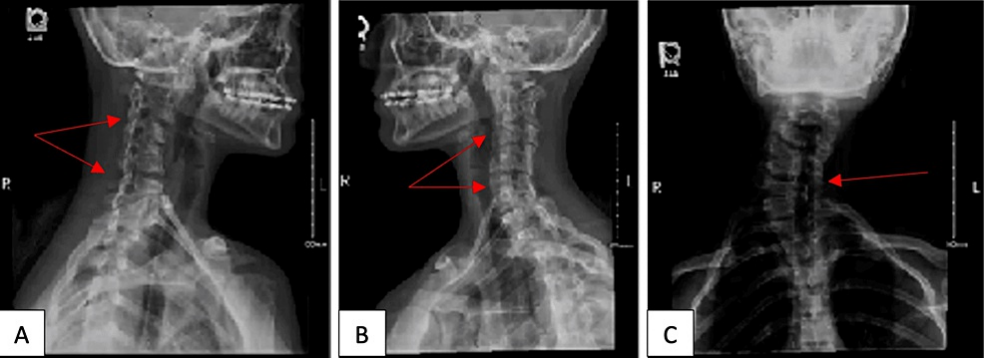
X-ray of the cervical spine (A) Partial fusion of C2-C5 and C6-C7 (arrows). (B) Partial fusion of C2-C5 and C6-C7 (arrows). (C) Scoliosis of the cervical spine and widening at C5-C6 (arrow).

This is a case report of an otherwise healthy 15-year-old female who presented to the otolaryngology outpatient clinic with complaints of a right-sided neck mass. The patient reported that she had noticed the mass about four months prior to the visit. She denied any changes in the growth of the mass since being first noticed, as well as having any upper respiratory infections or having recently been on antibiotics. The patient also denied neck stiffness. A review of symptoms for the patient was negative, including any symptoms of headaches, vision changes, or hearing changes. Family and social histories were reviewed and were non-contributory. Physical examination revealed pain on turning of the neck with an obvious, palpable, firm prominence at the base of the neck on the right side at level V (posterolateral), as well as a pronounced curvature of the cervical spine. The remainder of the patient's head, ear, nose, and throat examination was unremarkable. The patient was sent for a cervical spine X-ray. A review of the right-sided neck mass showed a partial congenital fusion of C2-C5 and C6-C7 with a C5-C6 disc widening, findings consistent with Klippel-Feil syndrome, in addition to findings of cervical scoliosis (Figure 1). No other imaging was done at this time.

## Discussion

The congenital cervical spinal fusion characteristic of Klippel-Feil syndrome is a result of a failure in the proper segmentation process that occurs between the third and eighth weeks of embryological development [1,2,5,7,8]. Mutations in a variety of genes have been implicated in Klippel-Feil syndrome, including those in growth differentiation factor 3 (*GDF3*) and *GDF6* and mesenchyme homeobox 1 (*MEOX1*) [2-4]. These genes play roles in the development, maturation, and differentiation of joints, cartilage, and bone during the embryological period [2,3]. The different mutations associated with Klippel-Feil syndrome lead to variations in inheritance patterns, and the Online Mendelian Inheritance in Man lists four different forms of KFS. Forms 1 and 3 with *GDF* mutations are inherited in an autosomal dominant manner, while the *MEOX1* mutation seen in form 2 and the myosin XVIIIB (*MYO18B*) gene mutation in form 4 are inherited as autosomal recessive [1-4,11-14].

As a result of the various genes implicated in this syndrome, patients may present with a wide range of clinical symptoms affecting multiple systems throughout the body. Most commonly, 60% of patients with KFS were found to also have scoliosis, and 35% had concomitant genitourinary tract abnormalities, including agenesis, ectopia, malrotation, dysgenesis, and duplication, with unilateral renal agenesis being the most common anomaly [5,8]. Musculoskeletal anomalies also include Sprengel's deformity (elevated scapula), seen in 20%-30% of patients [1-3,5].

Klippel-Feil anomaly may be a part of other syndromes, including Müllerian duct aplasia, renal agenesis, and cervical somite dysplasia (MURCS), Sprengel's deformity, and Wildervanck syndrome. Other commonly associated anomalies seen with Klippel-Feil syndrome included cardiac deformities, most commonly a ventricular septal defect, rib defects, facial and cranial asymmetry, and neurological complications such as hydrocephalus, syringomyelia, atlanto-occipital fusion, split cervical spinal cord, and meningomyelocele, which is the most common of the neural tube defects [1,5,6]. Clinically, patients with KFS are mostly asymptomatic, but when symptomatic, the most common complaints are of decreased cervical motion, as well as pain and neurological symptoms [2,7]. The diagnosis age is an important factor for early detection of KFS. Our patient presented with a non-enlarging neck mass with pain in cervical spine motion but denied any symptoms of neurological complications. Patients with KFS, however, are at an increased risk of cervical spine degeneration and should be monitored closely for the development of neurological signs and symptoms [3].

The diagnosis of KFS is made through cervical spine imaging showing fusion of the cervical vertebrae. Complete fusion of the vertebrae is associated with the wasp-waist sign, a radiographic appearance that occurs due to the fusion of cervical vertebrae, best visualized on lateral or sagittal imaging [3,5,10,15]. The clinical classification of KFS is based on the anatomical distribution of the fused vertebrae, of which three types have been described. Type 1 shows extensive cervical spine fusion and may also present with upper thoracic fusion. Type 2 is characterized by one or two cervical segment fusions, most commonly C2-C3 or C5-C6. Type 3 patients will have cervical spine fusion along with lumbar or lower thoracic fusion. Type 3 patients are often associated with other organ anomalies [1,3-5,8]. These specific findings help to differentiate KFS from other cervical region malformations such as ankylosing spondylitis, juvenile idiopathic arthritis, surgical fusion, and Chiari malformations.

Treatment is aimed at reducing the neurological sequelae of the syndrome and is based on the severity of the symptoms. Patients presenting with neurological symptoms should be referred for evaluation by neurosurgery [6]. In any case, patients with Klippel-Feil syndrome should be advised to avoid activities that may increase the risk of neck trauma, as these patients have been shown to have an increased risk for spinal cord injury, even with minor trauma [2,3,8,10]. Due to the increased association of renal anomalies in patients with Klippel-Feil syndrome, coupled with the increased incidence of renal stones in patients of any kind with spinal cord injury, KFS patients who withstand spinal cord injury are especially at risk of developing complications from difficult to treat nephrolithiasis, most notably renal failure [8]. As such, proper patient education is crucial to preventing complications and significant morbidity in patients with KFS. With appropriate education, precautions, and appropriate neurosurgery consultation when needed, the prognosis for a patient with Klippel-Feil syndrome is good [2].

Although a rare disorder, recent retrospective review studies of cervical spinal imaging have reported an incidence between 0.0058% and 0.02%, while Moses et al. found a prevalence of 1.2% within a pediatric population study [3,10]. These studies suggest that the prevalence of Klippel-Feil syndrome might be higher than previously reported and could warrant increased monitoring.

## Conclusions

Klippel-Feil syndrome is a rare disorder characterized by the classic triad of limited range of motion of the head and neck, short neck, and a low posterior hairline. Here, we described a case in which a 15-year-old female presents with a neck mass and pain in neck movement. The cervical motion restriction seen in patients with the syndrome is associated with an increase in degenerative changes of the spine and hypermobility of the non-fused segments. Although our patient did not present with any neurological signs, patients with KFS are at an increased risk of developing spinal cord compression, and even minor trauma can be quite problematic for these patients. This highlights the importance of early diagnosis and appropriate patient education.

Otolaryngologists, along with all primary care physicians, ought to keep Klippel-Feil syndrome on their list of differential diagnoses when working up a neck mass, as the classic triad is present in less than 50% of patients. In addition to the avoidance of high-risk activities, patients should also be sent for evaluation and workup to exclude possible renal and neurological anomalies commonly associated with KFS.

## References

[1] Lagravère MO, Barriga MI, Valdizán C, Saldarriaga A, Pardo JF, Flores M (2004). The Klippel-Feil syndrome: a case report. J Can Dent Assoc.

[2] Frikha R (2020). Klippel-Feil syndrome: a review of the literature. Clin Dysmorphol.

[3] Moses JT, Williams DM, Rubery PT, Mesfin A (2019). The prevalence of Klippel-Feil syndrome in pediatric patients: analysis of 831 CT scans. J Spine Surg.

[4] Mahajan UV, Labak KB, Labak CM, Herring EZ, Hdeib AM (2021). Images in spine: a rare abnormal bony fusion. Cureus.

[5] Yuksel M, Karabiber H, Yuksel KZ, Parmaksiz G (2005). Diagnostic importance of 3D CT images in Klippel-Feil syndrome with multiple skeletal anomalies: a case report. Korean J Radiol.

[6] Alam M, Haq AU, Shah M, Haqqani U, Ullah S (2020). Klippel-Feil syndrome with auxiliary anterior cervical meningomyelocele and thoracic syringomyelia: a case report. Spine (Phila Pa 1976).

[7] Tracy MR, Dormans JP, Kusumi K (2004). Klippel-Feil syndrome: clinical features and current understanding of etiology. Clin Orthop Relat Res.

[8] Vaidyanathan S, Hughes PL, Soni BM, Singh G, Sett P (2002). Klippel-Feil syndrome - the risk of cervical spinal cord injury: a case report. BMC Fam Pract.

[9] Samartzis D, Kalluri P, Herman J, Lubicky JP, Shen FH (2016). "Clinical triad" findings in pediatric Klippel-Feil patients. Scoliosis Spinal Disord.

[10] Gruber J, Saleh A, Bakhsh W, Rubery PT, Mesfin A (2018). The prevalence of Klippel-Feil syndrome: a computed tomography-based analysis of 2,917 patients. Spine Deform.

[11] Amberger Amberger, J.S. KLIPPEL-FEIL SYNDROME 2 (2022). Klippel-Feil syndrome 2, autosomal recessive; KFS2. https://www.omim.org/entry/214300.

[12] O'Neill O'Neill, M.J.F. KLIPPEL-FEIL SYNDROME 1, AUTOSOMAL DOMINANT; KFS1 (2022). Klippel-Feil syndrome 1, autosomal dominant; KFS1. https://www.omim.org/entry/118100.

[13] Freedman SB, Xie J, Nettel-Aguirre A (2017). Enteropathogen detection in children with diarrhoea, or vomiting, or both, comparing rectal flocked swabs with stool specimens: an outpatient cohort study. Lancet Gastroenterol Hepatol.

[14] Kniffin Kniffin, C.L. KLIPPEL-FEIL SYNDROME 4, AUTOSOMAL RECESSIVE, WITH NEMALINE MYOPATHY AND FACIAL DYSMORPHISM; KFS4 (2022). Klippel-Feil syndrome 4, autosomal recessive, with nemaline myopathy and facial dysmorphism; KFS4. https://www.omim.org/entry/616549.

[15] V U, Czarniecki M, Tatco V (2023). Wasp-waist sign (spine). https://radiopaedia.org/articles/wasp-waist-sign-spine?lang=us.

